# The influence of choline treatment on behavioral and neurochemical autistic-like phenotype in Mthfr-deficient mice

**DOI:** 10.1038/s41398-020-01002-1

**Published:** 2020-09-18

**Authors:** Galila Agam, Zoe Taylor, Ella Vainer, Hava M. Golan

**Affiliations:** 1 Faculty of Health Sciences, Department of Clinical Biochemistry and Pharmacology and Psychiatry Research Unit, Ben-Gurion University of the Negev and Mental Health Center, Beer-Sheva, Israel; 2 Zlotowski Center for Neurosciences, Ben-Gurion University of the Negev, Beer-Sheva, Israel; 3 Faculty of Health Sciences, Department of Physiology and Cell Biology, Ben-Gurion University of the Negev, Beer-Sheva, Israel

**Keywords:** Autism spectrum disorders, Predictive markers

## Abstract

Imbalanced one carbon metabolism and aberrant autophagy is robustly reported in patients with autism. Polymorphism in the gene methylenetetrahydrofolate reductase (Mthfr), encoding for a key enzyme in this pathway is associated with an increased risk for autistic-spectrum-disorders (ASDs). Autistic-like core and associated behaviors have been described, with contribution of both maternal and offspring Mthfr^+/−^ genotype to the different domains of behavior. Preconception and prenatal supplementation with methyl donor rich diet to human subjects and mice reduced the risk for developing autism and autistic-like behavior, respectively. Here we tested the potential of choline supplementation to Mthfr-deficient mice at young-adulthood to reduce behavioral and neurochemical changes reminiscent of autism characteristics. We show that offspring of Mthfr^+/−^ mothers, whether wildtype or heterozygote, exhibit autistic-like behavior, altered brain p62 protein levels and LC3-II/LC3-I levels ratio, both, autophagy markers. Choline supplementation to adult offspring of Mthfr^+/−^ mothers for 14 days counteracted characteristics related to repetitive behavior and anxiety both in males and in females and improved social behavior solely in male mice. Choline treatment also normalized deviant cortical levels of the autophagy markers measured in male mice. The results demonstrate that choline supplementation even at adulthood, not tested previously, to offspring of Mthfr-deficient mothers, attenuates the autistic-like phenotype. If this proof of concept is replicated it might promote translation of these results to treatment recommendation for children with ASDs bearing similar genetic/metabolic make-up.

## Introduction

Autism spectrum disorders (ASDs) are characterized by impaired social interactions, abnormal communication and stereotypic behavior. Numbers of diagnosed children is significantly increasing in recent years. On the basis of the high-heritability index a genetic component in the etiology of ASDs is obvious^1^ and the phenotype variability among ASDs subgroups suggests interactions between susceptibility genes and environmental factors. Namely, the origin of the disorder may vary between different subgroups within the spectrum. Therefore, it is plausible that effective intervention should be personalized and adjusted to well-defined subgroups.

Among the genes robustly found to be associated with increased risk for ASDs is methylenetetrahydrofolate-reductase (Mthfr)^2–10^. Mthfr activity affects the one-carbon (C1)-metabolism. C1-metabolism includes the folic-acid (FA)-dependent and FA-independent pathways, converging to convert homocysteine to methionine. MTHFR is a key enzyme in the FA-dependent pathway; the FA-independent pathway utilizes choline or betaine to produce methionine and the methyl donor S-adenosyl-methionine (SAM). The frequency of MTHFR’s single-nucleotide polymorphism, 677 C > T, is markedly higher among ASDs patients and their mothers than in the general population^10^ and the efficiency of its enzyme product to convert methylenetetrahydrofolate to 5-methyltetrahydrofolate is reduced. The finding of an increased risk for ASDs among newborns to mothers with homozygote polymorphism MTHFR677TT genotype if an additional gene-variant in the C1-metabolism is present^9^ supports the notion of an additive effect of C1 metabolic enzymes in the in utero environment for abnormal development. Furtheremore, altered plasma levels of SAM, S-adenosyl-homocystein (SAH), homocysteine and methionine levels and inadequate choline and betaine levels in plasma of a subgroup of ASDs patients culminate in low C1-metabolism regardless of their genotype^2,11–13^. The latter abnormalities may reflect genetic variations or factors such as eating problems and gastrointestinal complications highly prevalent in children with ASDs^14–17^.

We recently demonstrated autistic-like core and associated behaviors in Mthfr-deficient mice, with contribution of both maternal and offspring Mthfr-genotype to the different behavioral domains^18^. Prenatal or early postnatal supplementation of methyl-donors (FA, betaine and choline) decreased the risk of these mice to present ASD-like behavior^19^. Similar effect of prenatal choline fortification was reported in BTBR T + tf/J (BTBR) mice modeling core autistic-like behavioral deficits^20^. In human subjects with MTHFR677TT (vs. 677CC) polymorphism, using the methyl-donor choline to preserve methyl-group homeostasis is metabolically preferable over FA. This was replicated in Mthfr-knockout (KO) mice^21–24^ raising the possibility that choline fortification is more adequate for the metabolic requirements of this genetic ASDs subgroup.

Studies in ASDs patients and in ASDs-like animal models strongly suggest that autophagy, a group of catabolic pathways responsible for clearing damaged organelles and regenerating metabolic precursors, is involved in the pathophysiology of these disorders. Studies in patients revealed (i) a significant increase in the burden of rare copy-number variations (CNVs) and deletions in ASDs with enrichment for disruption of genes in autophagy pathways^25^; (ii) decreased levels of the autophagosome marker, LC3-II, and increased levels of the autophagy substrate, p62, in postmortem temporal-cortex of ASDs patients^26^. In mice models it has been reported that female mice deficient in ambra-1, a positive regulator of beclin-1 (a principal player in autophagosome formation), exhibited ASDs-like phenotypes^27^ and in PTEN-KO mice suppression of ASDs symptoms and improved neuronal abnormalities occurred following mTOR (mammalian target of rapamycin, an inhibitory regulator of autophagy induction) inhibition^28^.

Up-until-now the majority of ASDs children are diagnosed only at the age of at least 2–3 years old^29–32^ and, therefore, earlier intervention is not an option. The present study was set to test whether choline fortification to young adult mice with low C1-metabolism due to Mthfr heterozygote-KO ameliorates their autistic-like behavior and affects autophagy markers.

Here we show that choline supplementation to adult offspring mice of Mthfr^+/−^ mothers for 14 days normalized the deviation observed both in their autism-related behaviors and in protein levels of autophagy markers found altered in maternal or offspring Mthfr-haploinsufficiency. If replicated, it might promote translation of these results to treatment recommendation for children with ASDs bearing similar genetic/metabolic make-up.

## Methods

### Mice colony and study design

Mthfr^+/−^ breeding-pairs on a Balb/cAnNCrlBR background were kindly provided by Prof. Rozen (McGill University, Montreal, QC, Canada)^33^ to establish our local colony. Mice were maintained on a 12:12 h light/dark schedule, with food and water provided *ad libitum*. All procedures were performed according to the guidelines of the Israeli Council for Animal Care and approved by the Animal Care and Use Committee of Ben-Gurion University of the Negev (protocol IL-10-03-18).

To assess the effect of maternal and offspring Mthfr^+/−^-genotype (Het) *vs*. Mthfr^+/+^-genotype (Wt), Wt and Het female mice were mated with Wt males to generate three groups: Wt-offspring from Wt-mothers (Wt-Wt, *n* = 20 males, 19 females), Wt-offspring from Mthfr^+/−^-mothers (Het-Wt, *n* = 8 males, 14 females) and Mthfr^+/−^-offspring from Mthfr^+/−^-mothers (Het-Het, *n* = 14 males, 19 females). Sample size was selected based on the biological variation expected. Variation was evaluated in similar experiments by studies performed in mice of the same colony^18,19^. 94 offspring, delivered in 24 litters were included in the study, not more than 2 pups per litter were included in an experimental group. Female mice were tested regardless of their estrus-cycle status. Mthfr^−/−^ mice on a Balb/cAnNCrlBR background are not viable.

### Mthfr genotyping

Mice were genotyped as previously described^33^ using polymerase chain reaction (PCR) amplification of DNA isolated from toe clips. Primers used were:

Sense-primer 1 (5′-GAAGCAGAGGGAAGGAGGCTTCAG-3′) in Mthfr exon 3

Sense-primer 2 (5′-AGCCTGAAGAACGAGATCAGCAG C-3′) in the neo^r^ gene

Antisense-primer 3 (5′-GACTAGCTGGCTATCCTCTCATCC-3′) in Mthfr intron 3.

### Choline treatment

At postnatal day 80–100 all mice first underwent a set of autistic-related behavioral tests (pre choline), then administered either 0.003% choline-supplemented drinking water (replaced twice a week) or ordinary drinking water for two weeks and during the second run of the tests (post choline). In the second run Wt-Wt mice were split into two subgroups: controls - received ordinary drinking water; choline-treated - received choline-supplemented water.

### Behavioral tests

Mice were exposed to tests selected to evaluate general and autism-associated behaviors. A week before the experiments, each mouse was placed in a separate cage to avoid social hierarchy effect on behavior and mice were handled daily for two min to adapt them to the experimenter’s presence. Each mouse underwent a single test daily between 12:00 and 18:00. Males and females were tested sequentially on the same day in separate sub-sessions to allow room ventilation and cleanup. The testing arenas were cleaned between trials with 70% ethanol. Experiments were videotaped and analyzed offline. Open-field and object-recognition tests were analyzed using “EthoVision 9” software (Noldus, Wageningen, Netherlands). Sociability, social-preference, marble-burying and nest-building tests were analyzed manually by an observer blind to the group of the mice.

#### Open-field

**Fig. 1:**
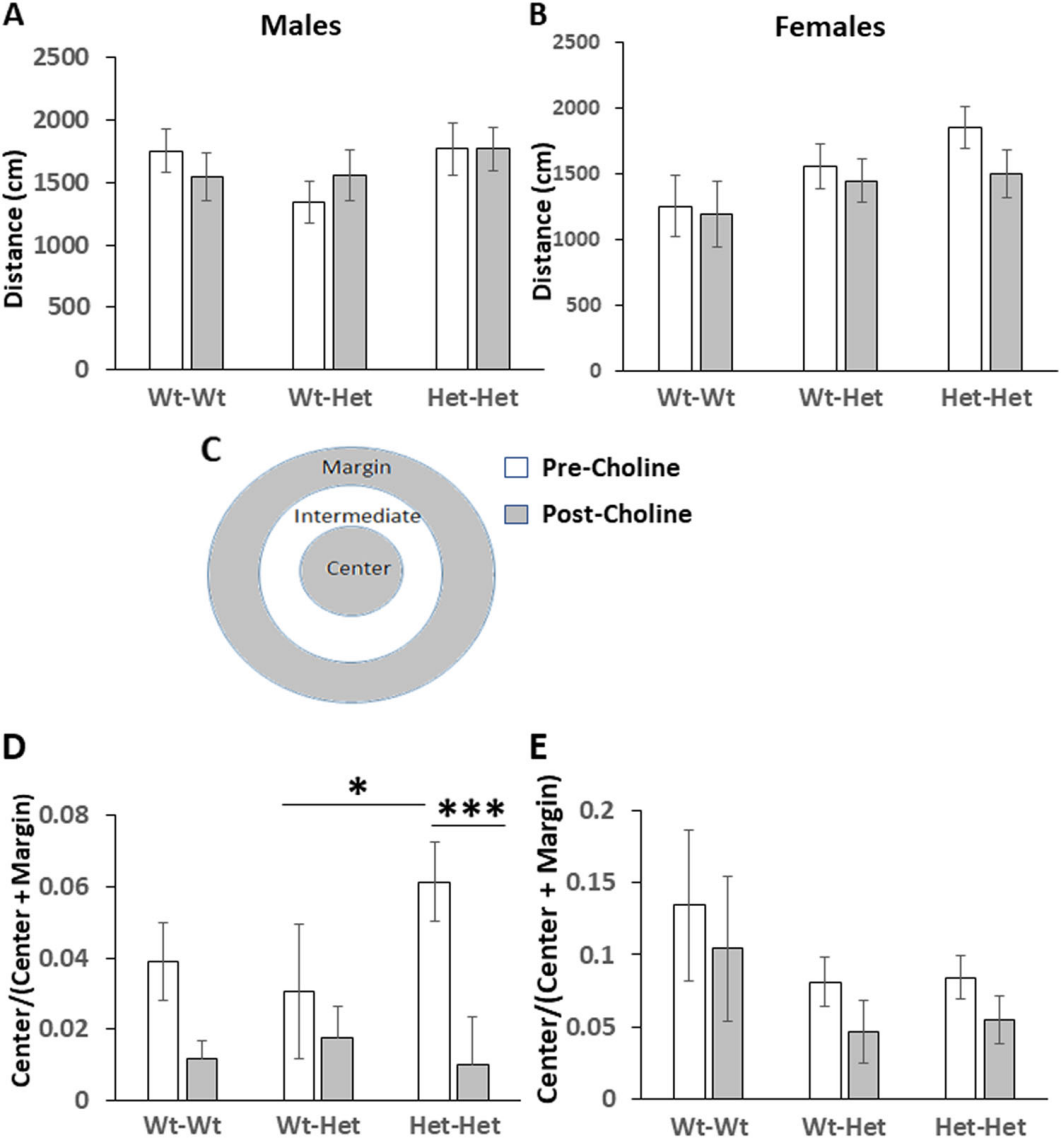
The effect of MTHFR genotype and choline on mice behavior in the open field. **a**, **b** Distance walked: **a** males; **b** females. **c** For offline analysis the area of the circular arena was divided into three sub-regions: center, intermediate zone and margins. **d**, **e** The ratio between the duration mice explored the center and the time of exploration of the center+margins was normalized to that of the Wt-Wt group: **d** males; **e** females. Results are means ± SEM; white bars—pre choline treatment, gray bars—post choline treatment; *N* = Male: Wt-Wt 19, Wt-Het 7, Het-Het 11; female: Wt-Wt 17, Wt-Het 12, Het-Het 17 for each condition. Results were analyzed by four-way ANOVA with the independent variables maternal Mthfr genotype, Mthfr genotype, treatment (±choline) and sex. **a**, **b** ANOVA: N.S. **d**, **e** main effects (males + females): maternal genotype, *F*_1,157_ = 5.34, *p* = 0.02; genotype, *F*_1,157_ = 3.23, *p* = 0.07; choline treatment, *F*_1,157_ = 6.495, *p* = 0.012; genotype × sex × treatment interaction, *F*_1,157_ = 8.06, p = 0.005; **d** male groups: genotype, *F*_1,53_ = 5.3, *p* = 0.026; choline treatment, *F*_1,53_ = 14.7, *p* = 0.000; genotype × choline interaction, *F*_1,53_ = 10.3, *p* = 0.002; *pre choline treatment the Het-Het mice exhibited a significantly higher center/center+margins ratio than the Wt-Het mice, and choline treatment counteracted this effect, Bonferroni post hoc test, *p < 0.05 and ***p < 0.001, respectively. **e** Female groups: maternal genotype, *F*_1,72_ = 5.6, *p* = 0.02.

General behavior, exploration, mobility and anxiety-related behaviors were tested in an open-field arena. Mice were placed for five min in a circular arena 55 cm in diameter surrounded by 20 cm-height walls^34^. Anxiety-index was calculated as the time a mouse spent in the center of the arena over the time it spent in the center+margins (Fig. 1); a shorter time in the center indicated higher anxiety.

#### Nest-building

Different features of the nest were used to evaluate animal welfare and repetitive-behavior^35,36^. Nest material comprised tissue paper folded into a fixed size of 5 × 7 cm placed in each cage. Size and quality of the nest were measured 24 h after the insertion of the bedding. Scores (0–3) were given for material processing, centralization and symmetry. The volume of the nest was calculated by length × width × height^18^.

#### Marble-burying

Repetitive-behavior was also evaluated by the marble-burying test^37^. Each mouse was placed in a cage with 15 green marbles organized in 5 × 3 rows for 10 min and then removed from the cage. Cages were photographed before mouse introduction into and after mouse removal from the cage. Number of buried marbles was counted.

#### Social-behavior

To test olfactory function, a pre-requisite for sociability, each mouse was placed at one end of its home-cage and the time it took the mouse to find a pea-sized piece of cheese hidden among the mouse’s feces located at the opposite end of the cage was measured.

Social-behavior was assessed by testing sociability and social-preference. Sociability was examined in a three-chambered apparatus comprised of a rectangular arena divided into three, equally-sized chambers (40 × 20 × 22 cm^38^) with slight adaptation, as previously described^34^. To avoid bias of environmental cues and illumination on the sides of the arena, the arena’s position in the room was arranged so that the same cues will be in both sides of the arena. The test was comprised of three test-phases: habitation, sociability and social-preference. The mice were first allowed to habituate for 10 min. Then they were put in the central chamber. During the sociability-test an unfamiliar adult mouse was sited in a box placed at the rear end of the left chamber while an empty box was placed in the rear end of the right chamber. The separators between the chambers were removed and the tested mouse allowed to freely explore all three chambers for 10 min. For the social-preference test the tested mouse was returned to the central chamber and an additional unfamiliar adult mouse introduced to the previously empty box. The duration the tested mouse spent sniffing each box was recorded.

#### Recognition-memory

To evaluate recognition-memory two identical rectangular objects (4 × 2 × 2 cm) were arranged at a fixed distance from each other in a 55 cm diameter circular arena and mice were allowed to explore for 15 min. The time spent exploring each object was recorded. Twenty-four hours later, the mice were tested for another 15 min session in the same arena with one of the objects remaining unchanged (familiar-object) while the other replaced by a novel one similar to the familiar in size but different in shape and color. The duration a mouse spent exploring the objects and the number of object-sniffing events were analyzed and compared^39^. Mice that did not explore any object were excluded.

### Western-blotting of autophagy markers

Total protein was extracted by sonication of cerebral cortex tissue for 10 s at 4 °C, 50% power capacity (Heat System Ultasonic INC) in 150 μl lysis buffer: 50 mM Tris-HCl pH 7.5, 1 mM EDTA, 1 mM EGTA, 50 mM NaF, 1% (V/V) β-mercaptoethanol, 1 mM PMSF, 1 μl protease inhibitors cocktail and 1 μl phosphatase inhibitors cocktail (all chemicals from Sigma-Aldrich, St. Louis, MO). After centrifugation at 10,000×*g* for 15 min at 4 °C the supernatant was collected and protein concentration determined spectrophotometrically using NanoDrop 2000 (Thermo Scientific, Waltham, MA). Western-blotting was performed according to a standard protocol used in our lab^40^ on 10% polyacryl-amid gel and transferred to PVDF membrane. Each sample was tested in duplicates of 10 and 20 µg/lane, to verify linearity. Primary antibodies and their dilutions in TBST (Tris-buffered saline supplemented with Tween-20) were: p62, ab91526–1:1500, Abcam, Cambridge Science Park, Cambridge, UK; Beclin-1, #3495–1:1000, Cell Signaling Technology, Danvers, MA; LC3, L7543–1:1000, Sigma-Aldrich, ibid. Secondary goat-anti rabbit antibody ab97051–1:10000, Abcam, ibid, diluted in TBST.

### Statistical analysis

Statistical analysis was performed using SPSS-23 software. Univariate three-way and four-way ANOVA was used to test the effect of and interaction between the independent factors of genotype, maternal genotype and sex. Mice from each sex were then tested separately for an effect of genotype and maternal genotype. One-way ANOVA with a Bonferroni post hoc test was used to compare the three genotype groups. ANOVA for repeated measurements was used to test the effect of and interaction between the independent factors of genotype, maternal genotype, sex and treatment. The effect of choline treatment was tested first on the Wt-Wt groups and thereafter on all choline-treated groups. Comparison of values measured in the chambers of the sociability arena were assessed by two-tailed or one-tailed paired student’s *t*-test for each group. When one-tailed test was used, it was indicated in the text. Results are presented as means ± standard error of mean (SEM). Values larger or smaller than mean ± 1.5 standard deviations (SD) were excluded. Differences with *P*-values ≤ 0.05 were regarded as significant.

## Results

We recently reported that mice heterozygosity of Mthfr-KO causes ASD-like behavior, with a significant contribution of both the offspring and the maternal genotype^18^. Using a similar phenotyping protocol the possibility to normalize behavior in young adult offspring of Mthfr^+/−^ mothers by choline supplementation was addressed.

### General locomotion, exploration, and anxiety

Absence of gross changes in mice behavior were confirmed by analyzing the total distance traveled in the open-field arena. As previously reported for non-treated mice^18^, males and females of all genotype groups demonstrated similar walking distance, ruling-out hyperactivity. The same result was obtained post choline treatment (Fig. 1a, b). Similarly to our previous observation^18^, prior to choline treatment, male Het-Het mice exhibited a lower anxiety-like phenotype, *p* = 0.026 (Fig. 1d) while both female offspring groups with maternal Mthfr-deficiency exhibited a higher anxiety-like phenotype, *p* = 0.02 (Fig. 1e). It should be noted that each mouse (except one group of the Wt-Wt mice) was tested prior to and post choline treatment. As could be expected, the values obtained in the second run (post-choline test) were 2- and 13-fold lower for males and females, respectively, as reflected by the Wt-Wt group (Wt-Wt-control) that were tested untreated in both runs. Intersession habituation in the open-field test is a well-established phenomenon reflecting adaptation and memory of the previous session^41^. For the sake of the statistical analysis and presentation all post-choline results were normalized by this factor. Choline treatment significantly decreased the center time-proportion of all male groups, *p* = 0.002. For detailed statistical analysis see figure legend.

### ASD-like behaviors

A set of tests representing autistic-like behavior reminiscent of core ASDs symptoms was used.

#### Repetitive-behavior

Repetitive-behavior was assessed by two tests representing different aspects of this behavior—marble burying and nesting material-processing^42^.

#### Marble-burying test (Fig. 2a, b)

Similarly to our previous report^18^, male and female Het-Het mice buried ~30%, *p* < 0.01, and ~50%, *p* < 0.05, respectively, more marbles than the Wt-Wt group. As for the effect of choline, there was an interaction between choline treatment and genotype. Choline significantly reduced the number of marbles buried by the male and female Het-Het mice, *p* < 0.001 and *p* < 0.05, respectively, in a manner that choline treatment counteracted the differences between the Het-Het mice and the Wt-Wt group. For detailed statistical analysis see figure legend.

**Fig. 2:**
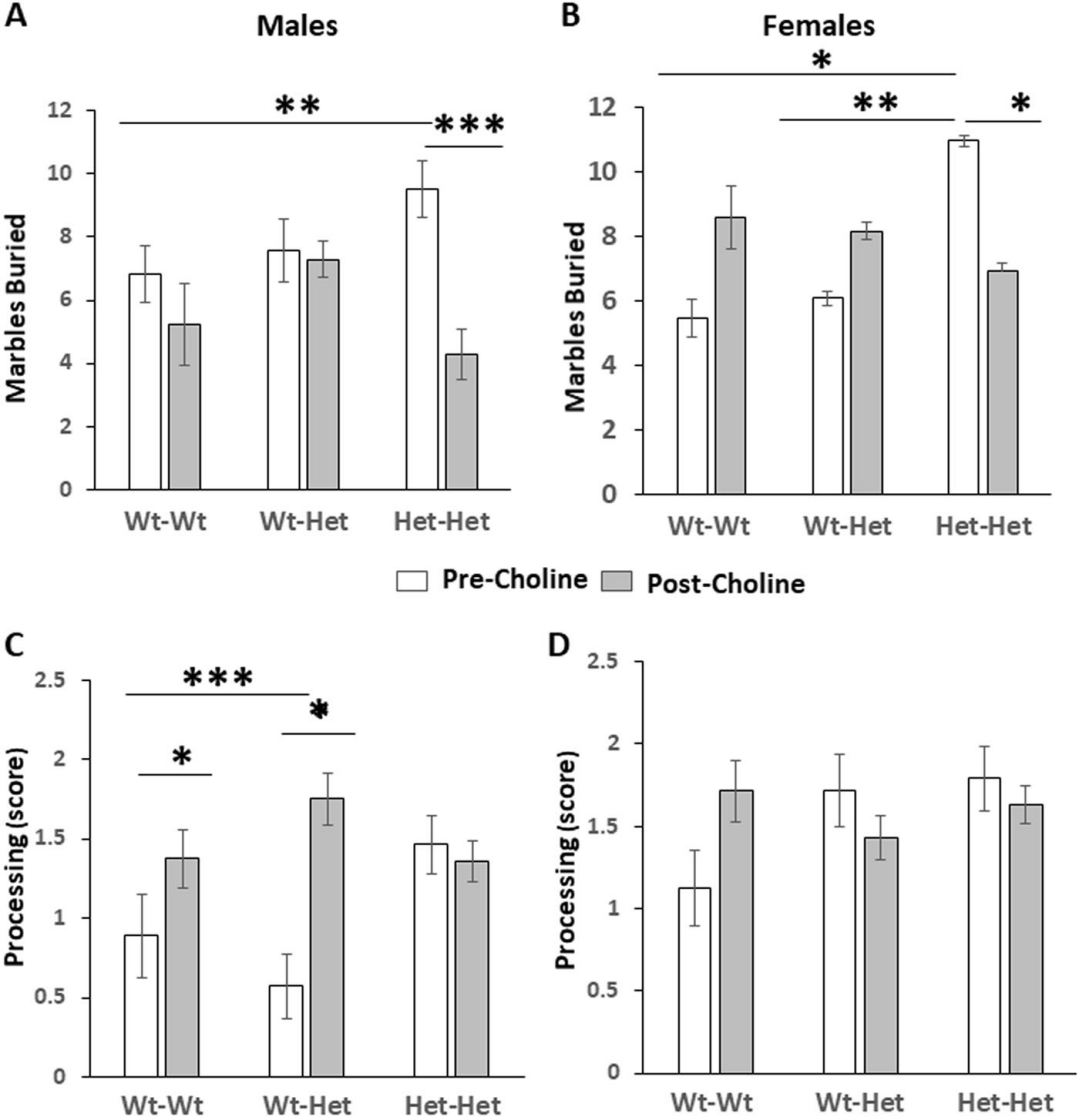
Choline alleviated repetitive behavior induced by MTHFR deficiency. **a**, **b** Number of marbles buried in the marble burring test. **a** Males; **b** females. **c**, **d** Nesting material processing score 24 h after introduction of new nesting material to the homecage. **c** Males; **d** females. Results are means ± SEM; white bars—pre choline treatment, gray bars—post choline treatment; *N* = Male: Wt-Wt 19, Wt-Het 7, Het-Het 14; female: Wt-Wt 17, Wt-Het 13, Het-Het 17 for each condition. Results were analyzed by 4-way ANOVA with the independent variables maternal Mthfr genotype, Mthfr genotype, treatment (±choline) and sex. **a**, **b** Main effects (males + females): choline treatment, *F*_1,173_ = 6.5, *p* = 0.012; genotypeXcholine treatment interaction, *F*_1,173_ = 16.5, *p* = 0.000. **a** Male groups: choline treatment, *F*_1,74_ = 6.5, *p* = 0.001; genotypeXcholine treatment interaction, *F*_1,74_ = 5.9, *p* = 0.017; pre choline treatment the Het-Het mice buried significantly more marbles than the Wt-Wt mice and choline treatment counteracted this difference, Bonferroni post hoc test, **p < 0.01 and ***p < 0.001, respectively. **b** Female groups: genotype, *F*_1,98_ = 4.5, p = 0.035; genotypeXcholine treatment interaction, *F*_1,98_ = 12.5, *p* = 0.001; pre choline treatment the Het-Het mice buried significantly more marbles than the Wt-Wt and the Wt-Het mice, *p < 0.05 and **p < 0.01, respectively, and choline treatment counteracted these differences, Bonferroni post hoc test, *p < 0.05; **c**, **d** main effects (males + females): genotype, *F*_1,181_ = 8.5, *p* = 0.004; choline treatment, *F*_1,181_ = 15.3, *p* = 0.000; sex, *F*_1,181_ = 13.1, *p* = 0.000; sex × choline treatment interaction, *F*_1,181_ = 4.4, p = 0.04. **c** Male groups: genotype, *F*_1,77_ = 9.8, *p* = 0.003; choline treatment, *F*_1,77_ = 6.4, *p* = 0.01; pre choline treatment Wt-Het mice processed nest material less than the Wt-Wt group, Bonferroni post hoc test, ***p < 0.001 and choline treatment counteracted this difference, Bonferroni post hoc test, *p < 0.05; **d** female groups: choline treatment, *F*_1,103_ = 9.4, *p* = 0.003.

#### Processing of nesting-material (Fig. 2c, d)

Similarly to our previous report^18^, male but not female Wt-Het mice processed nest-material less than the Wt-Wt group, *p* < 0.001; choline treatment counteracted this difference, *p* < 0.05. Choline treatment had an overall significant effect on all female groups, *F*_1,103_ = 9.4, *p* = 0.003, so that it elevated processing in the Wt-Wt group and decreased it in the offspring of heterozygote dams. For detailed statistical analysis see figure legend.

### Social-behavior

Prior to testing social-behavior mice underwent the olfactory-function test to ensure it is intact. As we previously reported none of the groups demonstrated olfactory impairment^34,43^.

**Fig. 3:**
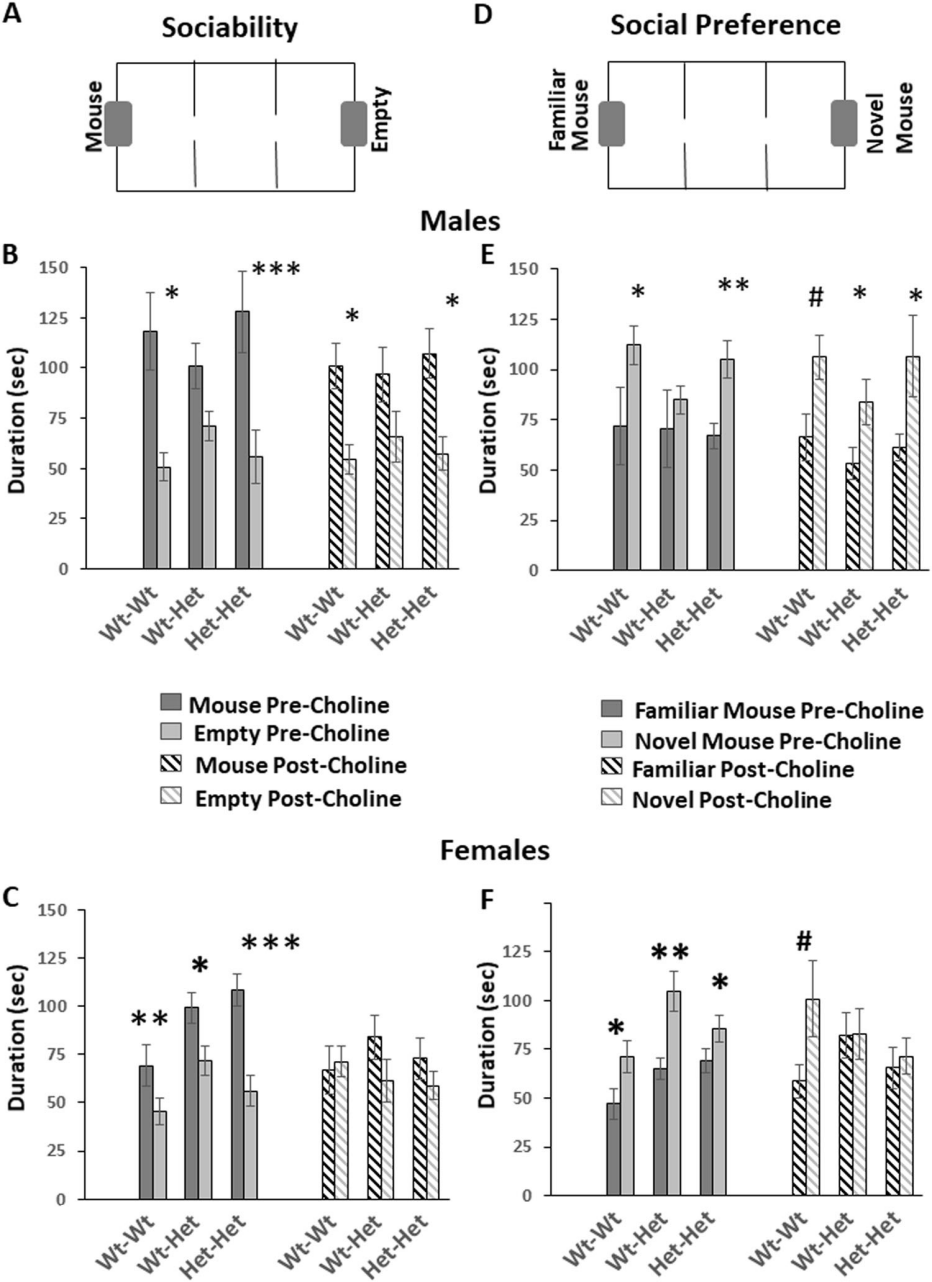
Choline treatment and MTHFR genotype affect the social behavior of mice. **a**, **d** A schematic illustration of the arena used to test social behavior. Note the dark gray rectangular shapes at the two distal walls of the arena, representing small boxes where **a**—sociability test—a mouse or nothing is located; **d** social preference test—an unfamiliar or a familiar mouse are located. **b** (male groups) and **c** (female groups)—sociability, evaluated as the time spent by a tested mouse sniffing the empty box vs. the box containing a mouse. **e** (male groups) and **f** (female groups)—social preference, evaluated as the time spent by a tested mouse sniffing the familiar mouse box vs. the novel mouse box. Results are means ± SEM; dark and light gray bars—pre choline treatment; dashed dark and light gray bars—post choline treatment;. *N* = Male: Wt-Wt 19, Wt-Het 8, Het-Het 11; female: Wt-Wt 18, Wt-Het 14, Het-Het 17 for each condition Results were analyzed by two-tailed paired *t*-test comparing the duration of sniffing between the two sides, with significant differences indicating preference for one of the sides. Choline treatment did not restore the impaired sociability of the Wt-Het male group, did restore this group’s social preference and abolished the sociability of all genotype female groups and the social preference of the maternal Mthfr^+/−^ female groups. *p < 0.05, **p < 0.01, ***p < 0.001, hash indicates one-tailed student’s *t*-test, *p* < 0.05.

In the *sociability-test*, where the mice had a choice between a small box holding a mouse vs. an empty box (Fig. 3a), prior to choline treatment, male Wt-Wt and Het-Het mice and all female groups exhibited a significant preference to explore the mouse, as reflected by longer duration spent sniffing the box holding the mouse vs. the empty box (Males: Wt-Wt, *p* = 0.02; Het-Het, *p* = 0.001; females: Wt-Wt, *p* = 0.002; Wt-Het, *p* = 0.033; Het-Het, *p* = 0.0001). Male Wt-Het group had no such preference (Fig. 3b). Choline treatment did not restore the preference of the male Wt-Het group and did not abrogate that of the Wt-Wt and Het-Het male groups but abolished the preference of all female groups (Fig. 3c).

In the *social-preference test*, where a mouse is given the option to spend time with a familiar-mouse vs. a novel-mouse (Fig. 3d), prior to choline treatment, male Wt-Wt and Het-Het mice presented a significant preference towards the novel-mouse (*P* < 0.05 for both groups). The Wt-Het male group demonstrated no such preference. Choline treatment restored this aberrant behavior (*p* = 0.021); the two other male groups kept their preference to the novel-mouse (Fig. 3e). Prior to choline treatment all female groups preferred exploring the novel-mouse (Wt-Wt, *p* = 0.01; Wt-Het, *p* = 0.002; Het-Het, *p* = 0.05). Choline treatment abolished this preference in both maternal Mthfr^+/−^ female groups (Fig. 3f).

### Recognition-memory

**Fig. 4:**
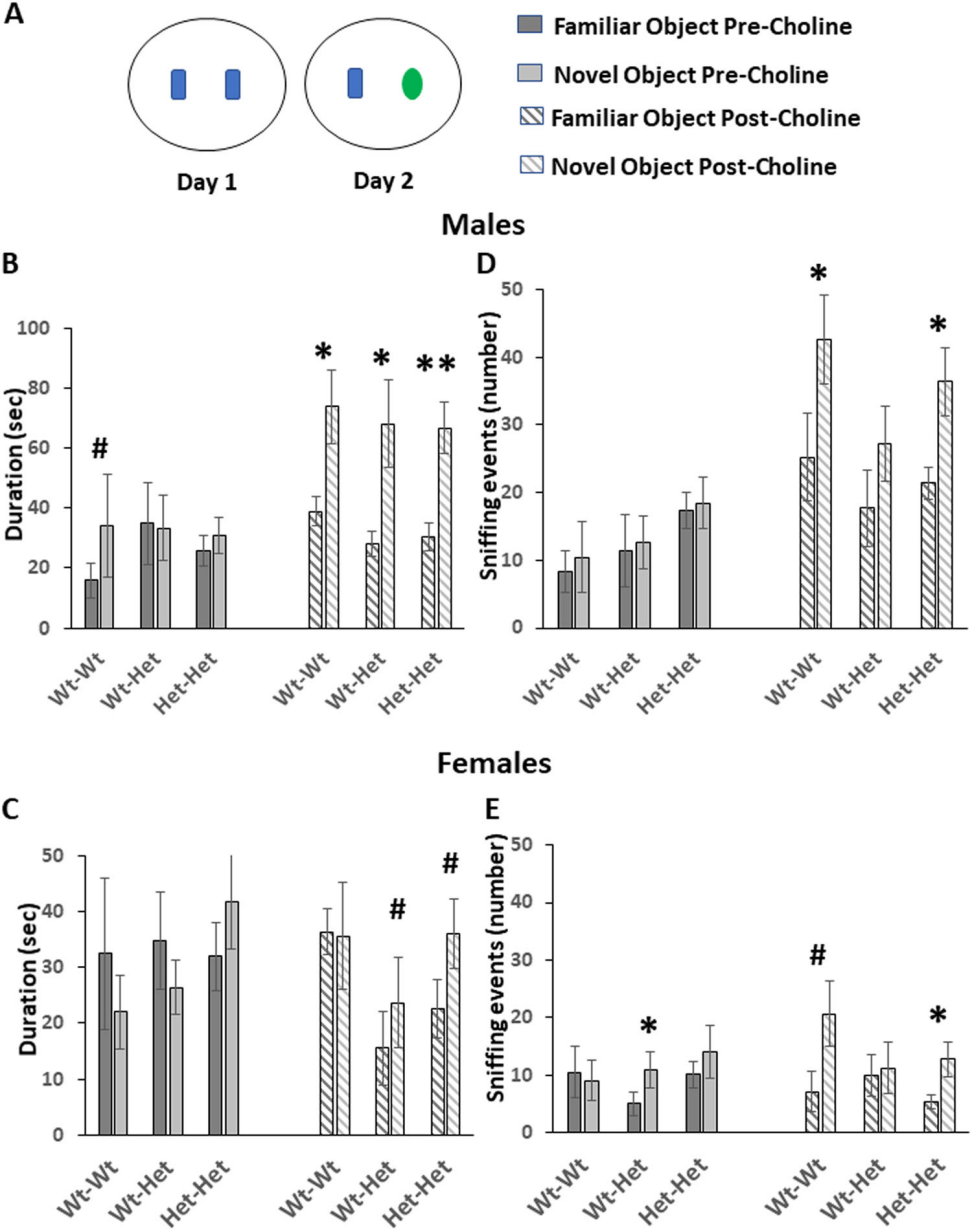
Choline improved performance of MTHFR-deficient mice in the object recognition task. **a** A schematic illustration of the object recognition arena on the first and second days of the test. **b**, **d** (male groups), **c**, **e** (female groups)—the duration (**b**, **c**), and the number of times (**d**, **e**) the tested mice spent sniffing each object on the second day of the test. Results are means ± SEM; dark and light gray bars—pre choline treatment; dashed dark and light gray bars—post choline treatment; *N* = Male: Wt-Wt 19, Wt-Het 8, Het-Het 14; female: Wt-Wt 17, Wt-Het 14, Het-Het 17 for each condition. Results were analyzed by two-tailed paired *t*-test comparing the duration and frequency of sniffing the novel object vs. the familiar one with significant differences indicating preference for one of the objects. The lack of preference, duration-wise, of the male and female Mthfr-deficient mice for the novel object was restored by choline treatment. Frequency-wise, prior to choline treatment, neither group demonstrated preference to the novel object. Choline treatment induced this preference in the Wt-Wt and the Het-Het groups. *p < 0.05, **p < 0.01, hash indicates one-tailed student’s *t*-test, *p* < 0.05.

As expected, on the first day of the object recognition test, in which mice were presented with two similar objects, the groups did not differ in the duration spent exploring either object, ruling-out bias due to object location. On the second day of the test, when one object was replaced with a novel-object (Fig. 4a), unlike the maternal Mthfr^+/−^ male mice, the Wt-Wt male group showed a tendency to explore the novel-object for a longer time compared to the familiar-object (one-tailed student’s *t*-test, *p* = 0.04), indicating no preference of the Mthfr^+/−^-deficient mice for either of the objects. Choline treatment restored the preference of the novel-object of the latter groups, Wt-Het, *p* = 0.04, Het-Het, *p* = 0.001 (Fig. 4b). In female mice, prior to choline treatment, all groups lacked novel object preference. Choline treatment restored the preference of the Wt-Het and the Het-Het groups, one-tailed *t*-test, *p* = 0.03 and *p* = 0.045, respectively, Fig. 4c). As for the number of sniffing events, prior to choline treatment only the female Wt-Het group demonstrated preference to the novel-object (*p* = 0.02). Choline treatment induced a significant preference in the male Wt-Wt and Het-Het groups (*p* = 0.05 and *p* = 0.01, respectively), in the female Wt-Wt group (one-tailed *t*-test, *p* = 0.03) and in the Het-Het group (*p* = 0.03) (Fig. 4d, e).

### Autophagy markers (males)

**Fig. 5:**
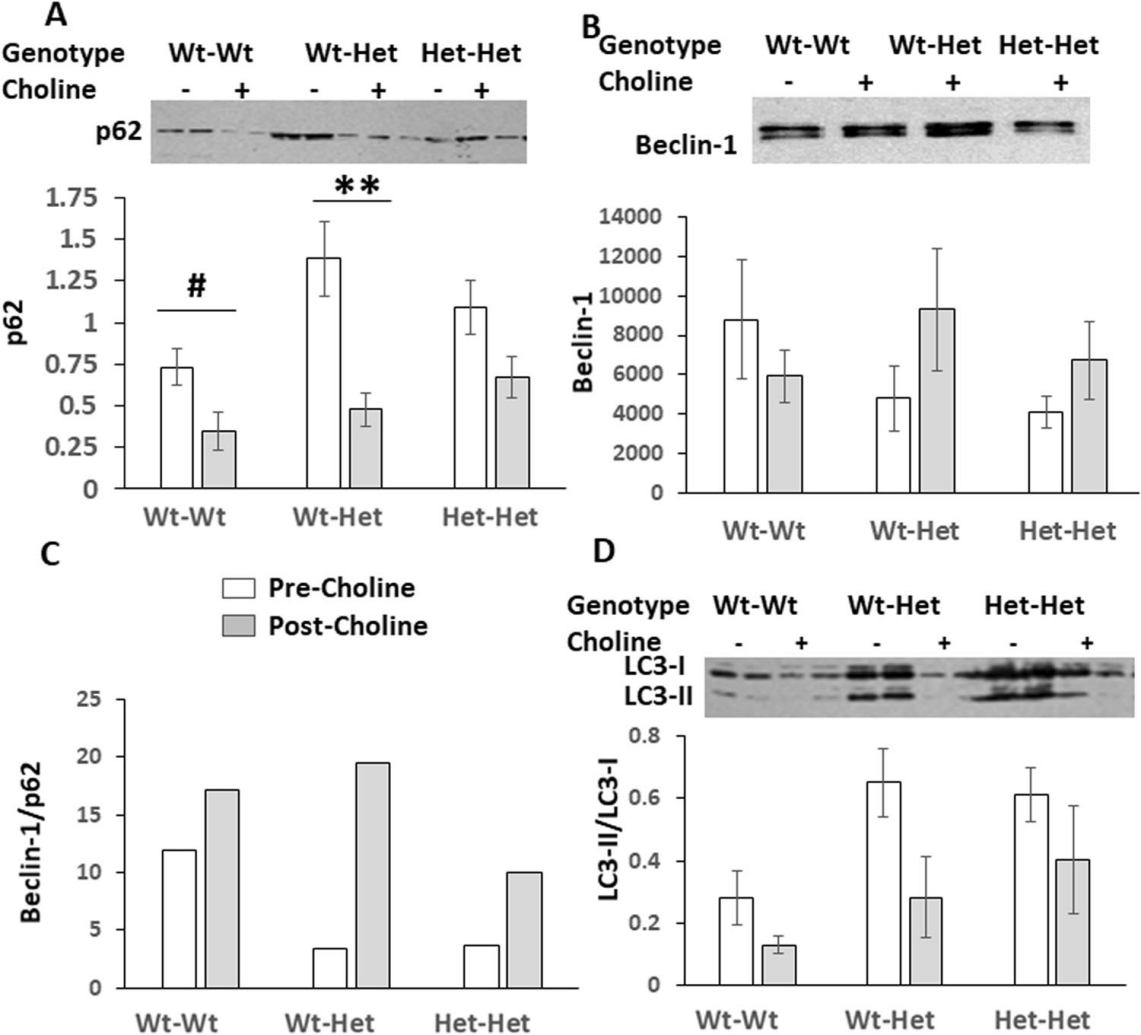
Choline treatment counteracts Mthfr insufficiency-induced cerebral cortex deviated protein levels of autophagy markers in male mice. **a** p62, a representative blot and means ± SEM densitometry of the intensity of the bands. **b** Beclin-1, ibid. **c** Beclin-1/p62 means’ ratios. **d** a representative blot of LC3-I and LC3-II and LC3II/LC3I ratio of their bands’ means ± SEM densitometric intensity in each sample. White bars—pre choline treatment; gray bars—post choline treatment. *N* = pre-choline: Wt-Wt 9, Wt-Het 4, Het-Het 5; Post-choline: Wt-Wt 9, Wt-Het 4, Het-Het 9. Results were analyzed by three-way ANOVA with the independent variables maternal Mthfr genotype, Mthfr genotype and treatment (±choline). p62—main effects: maternal genotype, *F*_1,36_ = 9.49, *p* = 0.004; choline treatment, *F*_1,36_ = 21.26, *p* = 0.000. Choline treatment reduced p62 levels of the Wt-Wt group, hash indicates one-tailed student’s *t*-test, *p* < 0.05 and significantly counteracted the elevated levels of the Wt-Het group, Bonferroni post hoc test, **p < 0.01. LC3-II/LC3-I ratio - main effects: maternal genotype, *F*_1,36_ = 4.227, *p* = 0.052; choline treatment, *F*_1,36_ = 5.08, *p* = 0.035. Namely, choline treatment significantly counteracted the elevated ratios of both Mthfr-deficient groups.

To find out whether Mthfr-haploinsufficiency recapitulates previously reported aberrant brain cortex autophagy in ASDs patients and ASD-like animal models^25–28^ and whether choline treatment is beneficial to neutralize potentially deviated autophagic homeostasis, frontal-cortex protein levels of three autophagy markers—p62, Beclin-1, and LC3, were measured (Fig. 5). p62 levels (Fig. 5a) were increased about two- and 1.5-fold in the Wt-Het and the Het-Het groups (ANOVA for the effect of maternal genotype: *F*_1,37_ = 7.442, *p* = 0.010). Beclin-1 levels (Fig. 5b) in the Wt-Het and Het-Het groups were non-significantly reduced by ~50% compared to the Wt-Wt values. We have previously established the Beclin-1/p62 ratio as an accepted indicative measure of autophagy efficiency^44–46^. Since values for both proteins in given mice were only available for a scarce number of animals we plotted the ratio of the means (Fig. 5c). Wt-Het and Het-Het mice exhibited about half the ratio size compared with that of the Wt-Wt mice, indicative of reduced autophagy efficiency. On the other hand, the LC3-II/LC3-I ratio, a measure of autophagosome formation, was elevated in the Wt-Het and the Het-Het groups (ANOVA for the effect of maternal genotype: *F*_1,27_ = 4.227, *p* = 0.05). Choline treatment tended to normalize the levels of all three autophagy markers/ratios towards those of the Wt-Wt group (Fig. 5d). Namely, choline significantly reduced all groups’ p62 levels and LC3-II/LC3-I ratios (ANOVA for choline treatment effect: *F*_1,37_ = 19.802, *p* < 0.001 and *F*_1,27_ = 5.081, *p* = 0.035, respectively). Beclin-1 levels of offspring of Mthfr-heterozygote mothers showed a non-significant trend of increase by choline compared to their prior levels. Choline treatment also normalized Beclin-1/p62 ratio of both Mthfr-deficient groups.

## Discussion

The present study builds on our previous reports^18,19^ of autism-like characteristics of offspring to mothers with heterozygote Mthfr-deficiency. The aims of the present study were (a) to substantiate these mice as an animal-model of some of the facets of autistic-like behaviors and neurochemistry, and (b) to expand the study and investigate, in view of the role of MTHFR in the C1-metabolism, whether choline treatment might be beneficial in amending such aberrant parameters.

As for the first aim, by-and-large, we replicated our previous reports of sex-dependent autistic-like behavior in offspring of Mthfr^+/−^ mothers, even if the genotype of the offspring was Mthfr^+/+^. Aberrant behavior in both groups of mice supports the long-lasting effect of the maternal genotype. Long-lasting effects of the intrauterine environment were described in relation with neurodevelopmental disorders, in general^9,47–49^, and in autistic-like models, in particular, such as the maternal immune-activation (MIA)- and valproate (VPA)-induced models^50,51^. The sex differences in the behavioral indices is not surprising. Although until recently the consensus in animal studies was the use of only males to avoid the effect of the estrus-cycle of females, it became obvious that sex differences exist on multiple levels including the phenotype of diseases^52^ and the response to treatment^53,54^. In particular, sex differences are well established in the autism field. Thus, the prevalence of autism as well as characteristics of the autistic phenotypes are different in males *vs*. females^55–58^. Similarly, differences between males and females in the performance in ASD-associated behavioral tests were found in the Mthfr-deficient mice and in other autistic-like mouse models^18,50–52,59,60^. Sex differences were also reported in the expression of several enzymes of the C1-metabolic pathway and in metabolite levels both regardless of the Mthfr genotype^23,61^ and in Mthfr-deficient mice^23,62^. These sex-related metabolic differences might underlie the female *vs*. male ASD-like behavioral disparities in the offspring of Mthfr^+/−^ mothers.

Replicated reports support the notion that autophagy is involved in the pathophysiology of ASDs^25,26,28,63^. This triggered us to expand the characterization of the Mthfr-deficient mice by evaluating cortical protein levels of autophagy markers. Our results prior to choline treatment, although only partially reaching statistical significance due to small sample numbers, imply increased p62 and reduced Beclin-1 protein levels in offspring with maternal Mthfr^+/−^ genotype (both Wt-Het and Het-Het) and drastically reduced (~50%) Beclin-1/p62 levels ratio. It is noteworthy that power analysis indicated that if twice the number of samples would have been available, the difference of Beclin-1 levels in the Wt-Het would have reached statistical significance. Beclin-1 is part of the autophagy-initiating PI3KC3 (phosphatidylinositol 3-kinase catalytic subunit type 3) complex which regulates autophagosome synthesis downstream the mTOR-independent pathway^64^. When autophagy is suppressed or disrupted the cargo carrier p62 accumulates. Hence, the Beclin-1/p62 ratio has been suggested by us, and accepted, as a coalesced marker indicative of autophagy efficiency^44–46^. Thus, our combined Beclin-1 and p62 levels and Beclin-1/p62 ratio results may be interpreted as deregulated autophagy in offspring of mothers with Mthfr^+/−^ and, as noted regarding the behavioral part of the study, the deviated levels of the autophagy markers in both groups of mice with maternal Mthfr-haploinsufficiency support the long-lasting effect of the maternal genotype. The results corroborate several previous reports in animal models with ASD-like characteristics as well as in ASDs patients as follows: Ambra^+/−^ female mice (Ambra is a Beclin-1 interactor that positively regulates autophagy)^64^ exhibit an autistic-like phenotype^26^; in Cc2d1a (coiled-coil and C2 domain containing 1A)^+/−^ mice, an animal model with ASD-like characteristics, hippocampal Beclin-1 expression levels were significantly decreased^65^; phospho-mTOR and p62 protein levels in postmortem temporal lobe of ASDs patients were higher than in control subjects^27^; similarly, a postmortem brain study found in a subset of ASDs patients increased p62 protein levels^66^. Apparently counterintuitively, and in contrast with Tang et al’s finding in the patients^63^, we found LC3-II/LC3-I ratio to be elevated in both groups with maternal Mthfr^+/−^ genotype. LC3-II, the lipidated form of LC3, is required for autophagosome formation and its upregulation is simplistically interpreted as indicative of elevated autophagy efficiency^67,68^. However, given that autophagy is a highly dynamic process modulated at several steps, increased amounts of LC3-II might also reflect accumulation of autophagosomes resulting from reduced autophagosome turnover^69^, such as in the case of inefficient fusion with endosomes and/or lysosomes, or due to inefficient degradation of the cargo once fusion has occurred^70^. If the latter is the case, then increased LC3-II/LC3-I ratio in the maternal Mthfr^+/−^ groups corroborates the reduced Beclin-1/p62 levels ratio and together they are indicative of deregulated autophagy in the cortex of these mice. Alternatively, increased LC3 lipidation might reflect induction of the LC3-associated phagocytosis (LAP) pathway^71^, corroborating with reports of enhanced or deregulated apoptosis in postmortem brain of subjects with autism and in other animal models of autism^72–74^. It has to be beard in mind that ASDs is not a single unique entity. Rather, it is a group of neurodevelopmental disorders with a similar behavioral phenotype. In addition, some well-characterized genetic syndromes also exhibit ASD-like characteristics. Interestingly, as was recently eloquently summarized by Magdalon et al.^75^, “*dysfunctional mTOR signaling has been identified as a molecular feature common to several syndromes with high prevalence of ASD*”. Nevertheless, as summarized by the authors, mTOR signaling is either hyperactivated or attenuated in the various ASD-related syndromes. The Mthfr-deficient mice do not, obviously, model all patient subgroups exhibiting an ASDs phenotype. Notably, in idiopathic autism patients, a significant downregulation of brain mTOR signaling was observed^76^. It is tempting to suggest that the Mthfr-deficient mice model this subgroup of patients, of whom the causative effect is yet unknown.

Choline plays a role in a myriad of cellular and metabolic functions spanning from cell membranes construction and signaling, methyl-group metabolism, lipid transport to neurotransmitter synthesis. Hence, it plays an important role in fetus brain and memory development^77,78^. Betaine, derived from dietary choline, is also a methyl-donor participating in re-methylation in the methionine cycle. It protects cells and proteins from environmental stress^79,80^. Choline supplementation has previously been reported to be beneficial in a variety of conditions, unrelated, indirectly- and directly-related to autism. Specifically, in human subjects bearing the Mthfr677C > T polymorphism and in Mthfr-deficient mice choline has been shown to be the preferred methyl-groups donor^22,23^. Until recently, choline supplementation was thought to be advantageous only in utero and was, therefore, studied during gestation. It was found beneficial both in human subjects^81^ and in rats in relation with social-behavior and memory in the context of the response to stress^82^. In Mthfr^+/−^ and BTBR mice choline was shown to reduce the risk of developing autistic-like behavior^19,20^. However, in the last couple of years, Jadavji et al.^83–85^ tested a combined supplementary diet including choline given at adulthood for its potential to protect or reduce impairment caused by ischemic damage. They report promising results such as improved motor function, reduced blood homocysteine levels, and elevated brain BDNF levels. Similarly, in the methyl-CpG-binding protein 2 (MECP2)-conditional KO mice modelling the genetic basis of Rett syndrome, a brain disorder with high prevalence of autistic features, choline supplementation was associated with improved object- and social-memory tasks along with enhanced complexity of dendrite arbor, spine density and synaptic activity^86^. A possible mechanism mediating choline’s effect is enhancing neuroprotective signaling and reducing oxidative stress^83,84^. In Jadavji et al.‘s study^84^ one of the direct causes for oxidative stress in the Mthfr-deficient mice might have been high homocysteine levels prevented by choline supplementation. It is conceivable that similar mechanisms were activated in the current study, promoting rectification of aberrant neurodevelopmental processes and reducing ongoing mutilation.

Based on the reports that (i) choline is a selective agonist of the brain α7 nicotinic acetylcholine receptor (AChR)^87^, (ii) abnormalities of the cholinergic system in postmortem brains of persons with ASDs, and (iii) the suggestion that the α7 nAChR in particular is a therapeutic target in ASDs (reviewed in Deutsch, and Burket, 2020)^88^, it may not be ruled out that another possible mechanism of, or contribution to, the behavioral effects observed following choline supplementation stem from choline’s role as an agonist of the α7 nAChR.

Choline is a substrate for the synthesis of phosphatidylcholine, a central component of neuronal plasma membrane. 1-oleoyl-2-palmitoyl-phosphatidylcholine (OPPC), an entity of phosphatidylcholine, is concentrated at the presynaptic area, playing a role in neuronal synapses in the brain^89^. On another level, by promoting de novo phosphatidylcholine synthesis, choline supplementation may affect the formation of autophagosomes^90^. Indeed, choline treatment normalized the levels of the autophagy markers towards those in the control group (Wt-Wt). This might have been a direct action or arbitrated by attenuation of oxidative stress through reduction of homocysteine levels^84^. In another malady-like condition accompanied by deregulated autophagy due to autophagosome accumulation, the ischemia-reperfusion model, choline was also found to exhibit a protective effect. Namely, it ameliorated deviated myocardial levels of Beclin-1, p62, and LC3 I/LC3 II ratio, apparently via activation of the mTOR signaling pathway^91^.

When choline treatment for neurodevelopmental disorders is considered, the question of treatment safety should be raised. The current study and those cited above found no adverse effects on control animals. Nonetheless, we do report now sex differences in the response to choline in the social tests, an aspect not tested before since solely male rodents were used in the experiments^78,83,84,86^. Given the basal female/male plasma and liver levels ratio of choline (range 1.4–2.75) and betaine (range 1.3–2.9)^61,92^, metabolites effective in supporting the C1-metabolism in a folate-independent pathway^23,24^, it is conceivable that in females, whose basic choline levels are high, choline supplementation dysregulates enzymes of this pathway or of others. This might explain our result in the social function-related tests, whereby choline treatment improved or had no effect in the males but worsened the females’ function. Hence, our study raises the need to adjust choline supplementation by sex, and, possibly, age. In this regard, to understand the origin and consequences of sex differences in C1-metabolism which might be important for precision medicine, Sadre-Marandi et al created a mathematical model of hepatic C1-metabolism based on physiological and biochemical female/male ratios of enzymes and metabolites of this pathway^93^. The model might be helpful in the potential application of choline supplementation, in general, and in females, in particular.

To summarize, offspring of Mthfr^+/−^ mothers exhibit several facets of autistic-like behavior and neurochemistry. Choline supplementation to adult mice for 14 days was sufficient to counteract some of the aberrant characteristics, mainly, repetitive-behavior and anxiety, both in males and in females. Improvement in other domains, such as social-behavior, was observed solely in the male mice. Deviant cortical protein levels of autophagy markers in the male mice were normalized by choline treatment.
